# Delivery of astragalus polysaccharide by ultrasound microbubbles attenuate doxorubicin-induced cardiomyopathy in rodent animals

**DOI:** 10.1080/21655979.2022.2050481

**Published:** 2022-03-24

**Authors:** Yanjie Liu, Li Chen, Hao Wu, Hebin Zhang

**Affiliations:** a Department of Ultrasound, The Second Affiliated Hospital of Zhejiang Chinese Medical University, Hangzhou, Zhejiang Province, China; b Department of Ultrasound, The Affiliated Hospital of Hangzhou Normal University, Hangzhou, Zhejiang Province, China

**Keywords:** Astragalus polysaccharide microbubbles, cardiomyopathy, oxidative stress, fibrosis, inflammation, AMPK signaling pathway

## Abstract

The aim of this study was to investigate the cardioprotective effects and probable mechanism of ultrasound-targeted microbubble destruction (UTMD) combined with astragalus polysaccharide (APS) on diabetic cardiomyopathy (DCM) model rats. The DCM rats with diabetes and cardiomyopathy were induced via chronic treatment of doxorubicin and then randomly divided into the (1) DCM model group; (2) APS microbubble group; (3) UTMDgroup; and (4) APS microbubbles combined with UTMD group. After 4-week intervention, the fasting blood glucose levels, body weight, %HbA1c level and glucose tolerance of DCM rats received combination therapy were significantly improved as compared with those of UTMD or saline-treated ones. Moreover, the heart/body weight ratio, and myocardial contractility were all improved after receiving combination therapy groups compared with others. In addition, significantly upregulated activities of superoxide dismutase (SOD) and glutathione peroxidase (GSH-Px) as well as significantly downregulated malondialdehyde (MDA) levels were all observed in the ones received combined treatment compared to others. Furthermore, the lipid accumulation and the expression levels of inflammatory factors were all significantly down-regulated in those ones received combination therapy compared with others (all *P* < 0.05). Further pathological analysis demonstrated that combination therapy effectively ameliorated fibrosis and myocardial morphological changes of DCM rats via activating the upregulation of AMPK and PPAR-γ signaling pathway, and inhibiting NF-κB activity in myocardial tissues of DCM rats. In conclusion, APS microbubbles combined with UTMD effectively protect the myocardial injury of DCM rats via activating AMPK signaling pathway to alleviate inflammation response, fibrosis and oxidative stress in myocardial tissues.

## Graphical abstract

**Figure:**
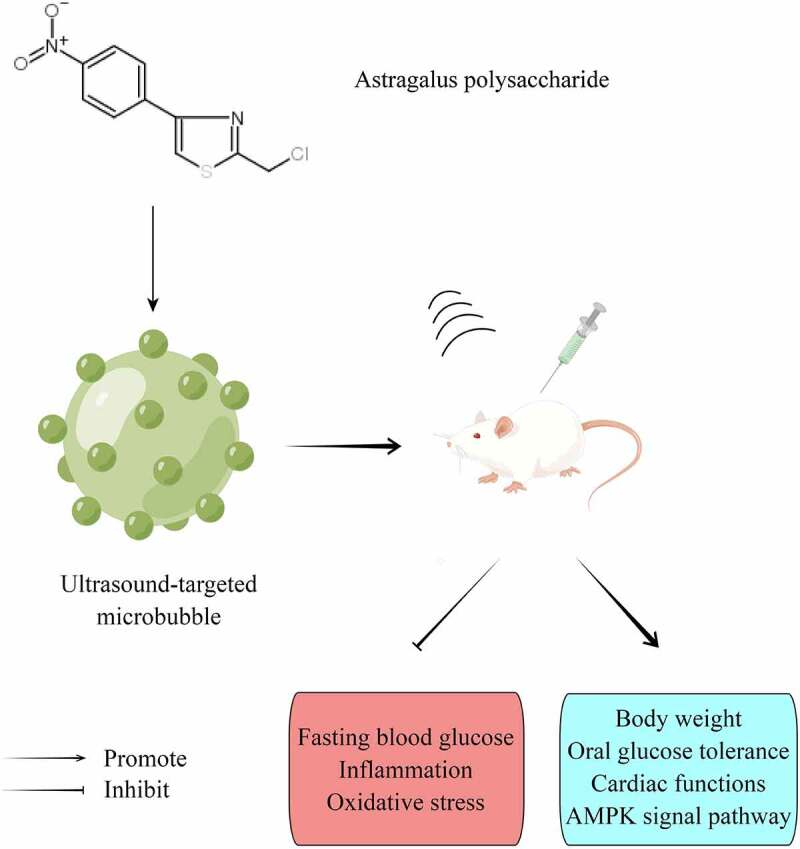

## 1. Introduction

It is estimated that diabetes is the third major category of disease in the world, and the clinical manifestations are mainly elevated blood glucose caused by absolute or relative deficiency of insulin [1,2]. Long-term diabetes can induce many complications, of which diabetes-related cardiovascular disease is the main cause of death [3]. Long-term insulin resistance (IR) and hyperglycemia can lead to a series of compensatory and decompensated reactions, and complex pathophysiological changes eventually lead to diabetic cardiomyopathy (DCM) cardiac structural changes, acting together on cardiac remodeling, and ultimately leading to damage to cardiac function [^3–5^]. Microvascular and macrovascular complications, as well as atherosclerosis-related diseases, are accompanied by other mechanisms such as hyperlipidemia and excessive inflammation [6]. This type of diabetic complication is formed by a disease process that directly affects the myocardium and is therefore defined as ‘diabetic cardiomyopathy’, independent of diastolic and systolic dysfunction other than coronary heart disease and other cardiac diseases [7]. In brief, diabetic cardiomyopathy is a serious complication of diabetes mellitus, which is defined as diastolic and systolic dysfunctions independent of coronary heart disease, hypertension and other heart diseases [3]. The pathogenesis of DCM is considered to be a non-myocardial cell remodeling caused by multiple factors of hyperglycemia and inflammatory response, leading to cardiac insufficiency, heart failure, shock and other symptoms [7]. It has been documented that the increase of oxidative stress is one of the main factors in the pathogenesis of DCM, which in turn is an important cause of myocardial fibrosis [8]. Although some treatments have been applied in clinical practice, the medical needs of DCM patients are still difficult to obtain a better satisfaction [9]. Therefore, how to effectively prevent and treat DCM has become a hot spot in clinical research, and active research and development of new drugs and methods that can effectively prevent and treat DCM has important clinical significance.

It is reported that the Astragalus polysaccharide (APS) has numerous pharmacological effects, including antihyperglycemic, antihypertensive, anti-viral, antioxidative and immune-enhancing activities [8,9]. Moreover, previous researches found that APS can suppress the NF-κB pathway, thereby inhibiting myocardial apoptosis produced after cardiac hemorrhage, suggesting that APS holds the potential to be a candidate molecule for improving myocardial injury [10,11]. Microbubble contrast agent, as a novel transport carrier, can carry drugs, cytokines, genes and other substances, burst microbubbles under certain energy ultrasound irradiation, and accurately release transport substances in target tissues, so as to achieve the purpose of targeted therapy [12,13]. At present, how to relieve diabetic myocardial injury is a medical problem that needs to be rapidly solved [7]. Studies at home and abroad have confirmed that some naturally occurring compounds, such as astaxanthin, have antioxidant activity and can improve the body’s immunity and prevent the occurrence and development of cardiovascular diseases, including myocardial injury and other diseases [14,15].

Given the benefit of APS for myocardial injury, we hypothesized APS-containing microbubbles that burst under ultrasound irradiation could increase drug concentration around myocardial tissue and protect myocardial tissue more effectively. Therefore, the aim of this study was to investigate the protective effect of ultrasound combined with microbubbles on myocardial injury in rats with diabetic cardiomyopathy and to explore the mechanism of regulating cardiac function and injury by detecting the levels of myocardial injury markers, oxidative stress and inflammation-related factors in serum and tissues, and histological section observation, providing new ideas and theoretical basis for the clinical treatment of DCM in the medical field.

## 2. Materials and methods

### 2.1 Materials

APS (purity >90%) was obtained from Tibobiochemistry Company (Beijing, China). The cellular apoptosis and ROS measurement kits were brought from Shanghai YoungChan Company (Shanghai, China). Trizol RNA extraction reagent, Real-timeScript™ reverse transcription kit, and SYBR oride were purchased from Sigma-Aldrich (USA). The SonoVue injection kit was purchased from BRACCO INTERNATIONAL BV (Spain). The antibodies targeting PPAR-γ, NF-κB, p-AMPK, t-AMPK, Beclin1 and Parkin were all brought from Abcam company (Cambridge, UK). Anti-mouse HRP secondary antibodies were also obtained from Abcam company (Cambridge, UK).

### 2.2 Preparation of APS microbubbles

According to the instruction of kit, 10 mL of 0.9% NaCl (injection grade) containing 1 mg APS were added to SonoVue vials, then vigorously shaken for 30 seconds until the completely dispersed lyophilized powders were observed, and incubated at 25°C for 120 min, during which several gentle oscillations were performed to obtain ultrasound microbubbles co-loaded with APS (SonaVue-APS).

### 2.3 Animal experiment

The GK rats in the model group received i.p. injection of adriamycin (2.5 mg/kg) once a week for 6 weeks. Once the modeling is done, APS (n = 6) and APS microbubbles + UTMD groups (n = 6) were administered via oral and tail i.v. injection at the dose of 0.5 mg/kg, while the UTMD alone group (n = 6) using the healthy SD rats as normal control received the same volume of saline solution. After the experiment, the precordiums of animals were shaved after being anesthetized by i.p injection of 10% chloral hydrate. The probe was placed in the rat precordium, and a short-axis view was taken at the level of the papillary muscle with a focal depth of 3.5–4.0 cm. When a large number of microbubbles were seen in the myocardium of rats, the microbubbles were repeatedly burst with the MBD function of the machine (mechanical index MI = 1.9) until they completely disappeared. Oral glucose tolerance tests were performed at a fixed time every week, as previously reported [16]. Fasting blood glucose and body weight of rats were also measured and recorded. The contents of TC and TG in serum were measured by an automatic blood biochemical analyzer. All the animal experiments were designed and performed according to the guidelines of the Ethics Committee on Laboratory Animals.

### 2.4 Evaluation of myocardial biochemistry and cardiac functions

Serum samples were obtained from rats before and every 2 weeks after intervention. Serum and myocardial total endothelin (ET) and calcitonin gene-related peptide (CGRP) levels were measured by radioimmunoassay. Serum levels of the CTnI, CK, LDH, SOD, MDA, ET and CGRP were detected by using ELISA kits in strict accordance with the instructions (Nanjing Jian Cheng Bioengineering Company). At the end of the intervention, the body weights were recorded and the rats were subsequently anesthetized by intraperitoneal injection of 2% sodium pentobarbital at 1 mL/kg. Subsequently, LVIDd, LVEDP and LVEF parameters of rats were immediately measured by M-mode ultrasound.

At the end of experimental period, the DCM rats in each group were sacrificed, followed by opening the thoracic cavity, clamping the ascending aorta and rapidly removing the heart, removing the great vessels of the rat heart, flushing with normal saline to remove the residual blood in the cardiac chamber, and sucking up the water with filter paper. All the hearts of the DCM rats were weighed on a microbalance to calculate the heart/body weight ratio (HW/BW). The right ventricles were further cut off, the left ventricle was preserved and part of the myocardium was cut perpendicular to the symmetrical midpoint of the long axis of the left ventricle of the heart and fixed in paraformaldehyde, embedded in paraffin 24 hours later, and serially sectioned for 5 mm. H&E staining and Masson staining were performed according to previously reported methods [17] and placed under a high-resolution light microscope to observe the morphological changes of myocardial tissue and the degree of myocardial fibrosis, and the CVF value was calculated using ImageProPlus 6.0 software. Histology scoring was performed by a blinded independent observer. The myocardial fibrotic area was calculated by the ratio of the positively stained fibrotic area to the whole area of the myocardium.

### 2.5 Western blot

After freezing and grinding of myocardial tissues, half of samples were added with protein lysates to extract protein and quantify protein via BCA method, followed by the western blotting method to detect protein expression of PPARγ and NFκB in myocardial tissue. Subsequently, the cardiac tissue protein samples were separated by 15% SDS-PAGE. The separated proteins were electro-transferred to PVDF membrane. Subsequently, the samples were further blocked by using the 5% BSA for 2 hours at 25°C, followed by incubating the primary antibody against different indicators overnight at 4°C. In addition, the PVDF membranes were developed using enhanced chemiluminescence after being incubated with HRP-labeled secondary antibody at the dilution of 1:4000. Furthermore, the PVDF membrane was further scanned and calculated via the Typhoon and Image J, respectively.

### 2.6 RT-qPCR

Myocardial tissues isolated from the rat heart were frozen and ground, half of which were added with protein lysate to extract protein and quantify protein, and the protein expression of PPARγ and NFκB in myocardial tissue was detected by western blot. In addition, the myocardial MDA content and SOD activity were measured by the xanthine oxidase method and the TBA method, respectively. The other half of the tissue was added with 1 mL of TRIozl reagent, repeatedly blown, aspirated and transferred into an EP tube, total RNA was extracted from the tissue according to the instructions of the kit, RNA quality and concentration were detected using a spectrophotometer, followed by reverse transcription and real-time PCR according to the instructions of TaKaRa reagent, in which the primers for β-actin and NF-κB were referred to the sequences used in previously published articles.

### 2.7 Statistical methods

Current result statistics were performed via using SPSS 13.0 software and showed as mean ± SD. The two independent samples *t* test was used for comparison. A value of *P* < 0.05 was considered statistically significant.

## 3. Results

1. Given the benefit of APS for myocardial injury, we hypothesized APS-containing microbubbles that burst under ultrasound irradiation could increase drug concentration around myocardial tissue and protect myocardial tissue more effectively. Therefore, the aim of this study was to investigate the protective effect of ultrasound combined with microbubbles on myocardial injury in rats with diabetic cardiomyopathy and to explore the mechanism of regulating cardiac function and injury by detecting the levels of myocardial injury markers, oxidative stress and inflammation-related factors in serum and tissues, and histological section observation, providing new ideas and theoretical basis for the clinical treatment of DCM in the medical field.

### 3.1 Chronic effects of combined APS microbubbles with UTMD on diabetic rats with DCM

**Figure 1.**
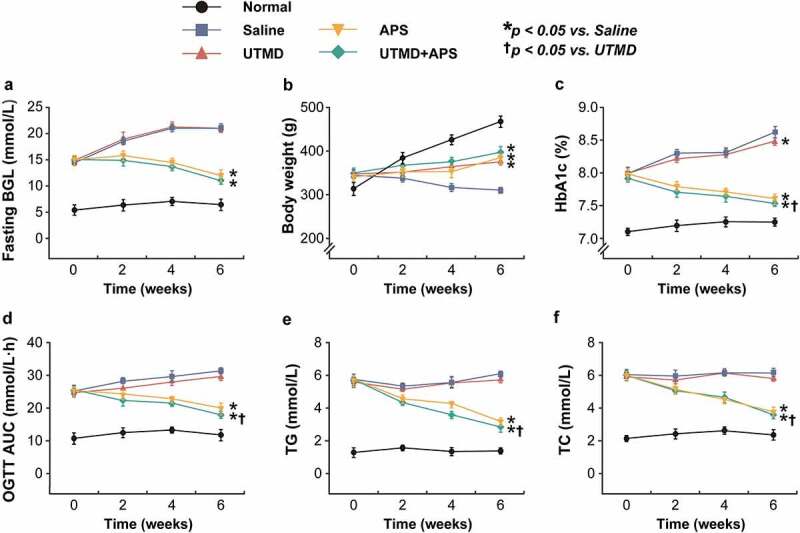
Chronic effects of combined APS microbubbles with UTMD on diabetic indexes of the GK rats with DCM. The (a) fasting blood glucose levels, (b) body weight changes, (c) %HbA1c level, (d) OGTT AUC, (e) TG and (f) TC levels of the GK rats with DCM. All results showed as Mean ± SD (n = 6). * *P* < 0.05 *vs*. Saline group; ^†^ *P* < 0.05 *vs*. UTMD alone group.

In this study, the protective effects of combined APS microbubbles with UTMD on diabetic indexes of GK rats were explored. As shown in Figure 1, the fasting blood glucose of the model rats received the saline or UTMD alone treatment continued to increase during the entire experimental period. In contrast, the blood glucose level was effectively controlled in the group that received the combination therapy, but the advantage over that of APS alone was not significant. Moreover, the body weight of GK rats in the model control group continued to decrease at 6 weeks of intervention, while there was no significant difference between the three intervention groups. The improvement in %HbA1c, glucose tolerance and blood lipid profiles in GK rats were all similar to the fasting BGLs, and the ones-received the combination treatment were significantly improved as compared with the control group, but not compared with APS alone group.

### 3.2 Chronic effects of combined APS microbubbles with UTMD on cardiac functions of diabetic rats with DCM

As the results shown in Table 1, conventional ultrasound results exhibited that the LvIDd and LVIDs in diabetic DCM rats were statistically significantly higher than those of the normal ones, while the LVFS and LVEF were significantly lower (both *P* < 0.05). After 6-week intervention, the downregulated or upregulated trend of LVEF/LVFS and LvIDd/LVIDs were obviously reversed in the group that received the combination therapy, while those in the UTMD or APS alone treated groups exhibited no significant difference compared with saline-treated diabetic rats with DCM, indicating the combined effects on the cardiac functions of diabetic rats with DCM.

**Table 1.** Chronic effects of combined APS microbubbles with UTMD on routine ultrasound cardiac functions of diabetic rats with DCM. All results are shown as mean ± SD (n = 8). * *P* < 0.05 *vs*. Saline group; ^†^ *P* < 0.05 *vs*. UTMD alone group; ^#^ *P* < 0.05 *vs*. APS alone group

Groups	LVIDs (mm)	LVIDd (mm)	LVEF (%)	LVFS (%)
Normal	3.96 ± 0.29	7.18 ± 0.34	82.77 ± 5.16	47.22 ± 3.58
Saline	5.23 ± 0.24	7.84 ± 0.21	58.52 ± 3.72	27.60 ± 3.09
UTMD	5.48 ± 0.36	7.63 ± 0.69	57.42 ± 4.87	27.81 ± 2.68
APS	4.75 ± 0.21 *	7.58 ± 0.42	69.06 ± 3.50 *	32.08 ± 3.16 *
UTMD+ APS	4.05 ± 0.19 *^,†, #^	7.07 ± 0.31 *	80.68 ± 5.36 *^,†, #^	42.58 ± 3.24 *^,†, #^

### 3.3 Chronic effects of combined APS microbubbles with UTMD on cardiac indicators of the DCM rats

**Figure 2.**
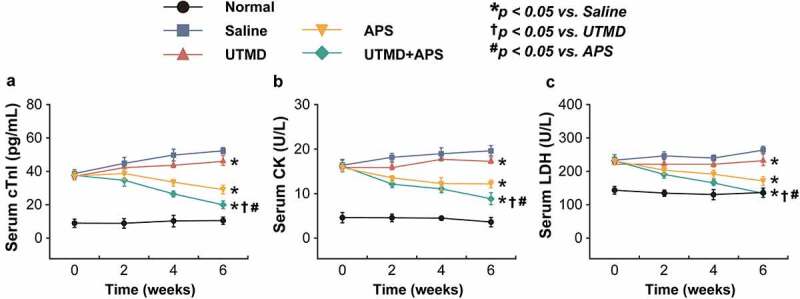
Chronic effects of combined APS microbubbles with UTMD on the cardiac indexes of diabetic rats with DCM. The serum (a) CK, (b) LDH, and (c) cTnl levels of the model rats. All results showed as Mean ± SD (n = 6). * *P* < 0.05 *vs*. Saline group; ^†^ *P* < 0.05 *vs*. UTMD alone group; ^#^ *P* < 0.05 *vs*. APS alone group.

Myocardial injury-related biomarkers in the rat serum were further detected. As showed in Figure 2, all three indicators, including cTnl, CK and LDH, of the saline-treated GK rats with DCM were significantly increased as compared with the normal group, while the combination group significantly reversed these increases of above indicators, and the improvement effect was very significant as compared with the two monotherapies, suggesting that the protective effects of combination therapy on the myocardial injury of DCM rats. Furthermore, as the results shown in Table 2, significantly increased ET and decreased CGRP levels in both serum and myocardium of the DCM rats treated with saline were observed (both *P* < 0.05). Significantly, these significant changes were all reversed in the group that received treatment of combined APS microbubbles (all *P* < 0.05).

**Table 2.** The contents of ET and CGRP in the rat serum and myocardium of the DCM rats. All results are shown as mean ± SD (n = 6). * *P* < 0.05 *vs*. Saline group; ^†^ *P* < 0.05 *vs*. UTMD alone group; ^#^ *P* < 0.05 *vs*. APS alone group

Groups		ET		CGRP	
		Serum (pg/mL)	Tissue (pg/g)	Serum (pg/mL)	Tissue (pg/g)
Normal rats	saline	179.2 ± 23.4	17.3 ± 2.5	97.3 ± 12.8	247.3 ± 52.1
DCM rats	Saline	286.3 ± 17.2	38.4 ± 8.5	21.4 ± 1.8	120.2 ± 15.2
	UTMD	251.7 ± 27.6 *	25.2 ± 6.9 *	45.2 ± 10.1 *	161.2 ± 21.8 *
	APS	219.8 ± 56.8 *	24.4 ± 5.3 *	54.6 ± 14.2 *	164.1 ± 13.4 *
	UTMD+ APS	197.2 ± 19.5 *^,†, #^	21.7 ± 7.5 *^,†, #^	72.5 ± 17.2 *^,†, #^	212.1 ± 31.4 *^,†, #^

Compared with normal healthy rats, the body weights of DCM rats in all groups were significantly decreased (all *P* < 0.05, Table 3). Significantly, the obviously reduced body weight of DCM rats was reversed via the chronic treatment of combined APS microbubbles with UTMD compared with that of the saline-treated DCM model group. Moreover, the heart weights of rats in the combination group were significantly reduced (*P* < 0.05) compared with the control group, while those of the other two monotherapies did not exhibit statistically significant difference (all *P* > 0.05). Furthermore, the Hw/Bw of the rats received treatment of combined APS microbubbles with UTMD group were significantly lower than that of all three other DCM rat groups (all *P* < 0.05), especially for the saline-treated model ones (*P* < 0.05).

**Table 3.** Chronic effects of combined APS microbubbles with UTMD on the body/heart weight ratio changes of diabetic rats with DCM. All results are shown as mean ± SD (n = 8). * *P* < 0.05 *vs*. Saline group; ^†^ *P* < 0.05 *vs*. UTMD alone group; ^#^ *P* < 0.05 *vs*. APS alone group

Groups		BW (g)	HW (mg)	HW/BW (mg/g)
Normal rats	Saline	468.56 ± 15.95	1042.33 ± 59.09	2.22 ± 0.15
DCM rats	Saline	339.29 ± 43.45	1205.32 ± 174.26	3.55 ± 0.35
	UTMD	345.86 ± 29.35	1221.26 ± 124.93	3.53 ± 0.26
	APS	419.08 ± 22.95 *	1191.99 ± 106.24	2.84 ± 0.27
	UTMD+ APS	428.46 ± 20.72 *^,†^	971.71 ± 94.08 *^,†^	2.27 ± 0.23 *^,†^

### 3.4 Chronic effects of combined APS microbubbles with UTMD on the cardiomymorphology of the DCM rats

**Figure 3.**
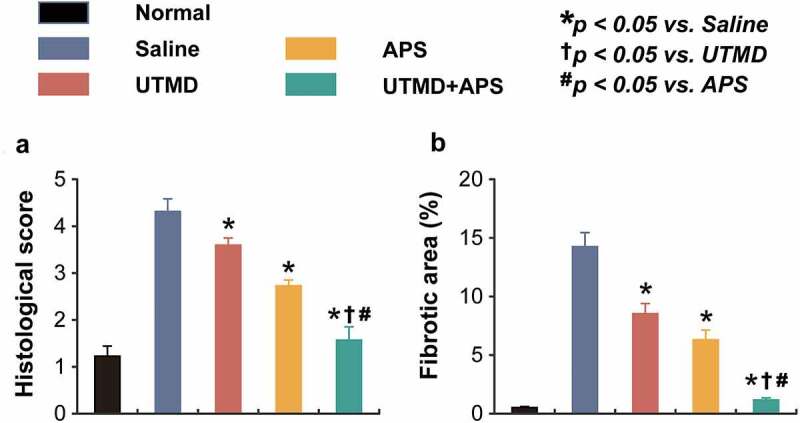
Chronic effects of combined APS microbubbles with UTMD on the cardiomymorphology of the DCM rats. (a) Histological score and (b) fibrotic area of heart tissues from the rats in different groups. All results showed as Mean ± SD (n = 6). * *P* < 0.05 *vs*. Saline group; ^†^ *P* < 0.05 *vs*. UTMD alone group; ^#^ *P* < 0.05 *vs*. APS alone group.

As the results of H&E staining analysis shown in Figure 3, the fibers of left ventricular myocardial tissues from the healthy rats were complete with clear boundary, and cardiac cells arranged regularly without significant lesion and infiltration of inflammatory cells. In contrast, the myocardial tissues of saline-treated DCM model rats were clearly damaged, showing as dissolved myofibrils, broken fibers and widened cell space. Significantly, the intercellular space was widened and a few myocardial fibers were broken and the fibers were relatively neatly arranged in the DCM group treated with combination therapy, indicating the potent cardioprotective efficacies of combined therapy on myocardial injury.

**Figure 4.**
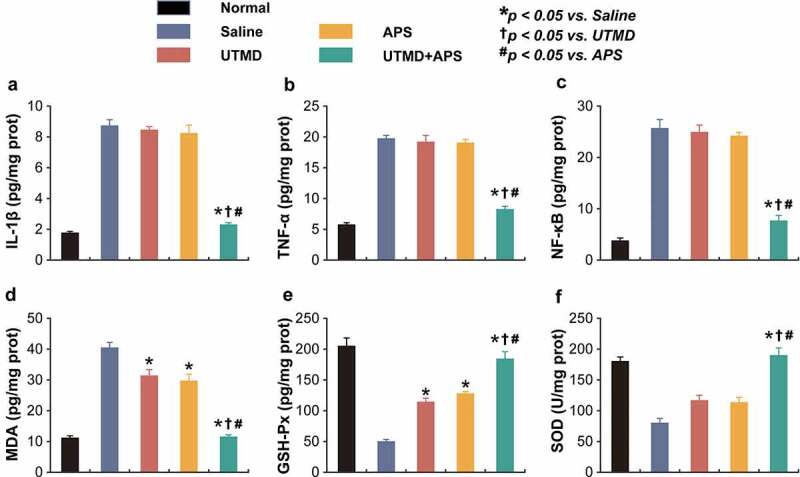
Chronic treatment of combined APS microbubbles with UTMD improves inflammation and oxidative stress in DCM rats. The levels (a) IL-1β, (b) TNF-α, (c) NF-κB, (d) MDA, (e) GSH-Px and (f) SOD. All results showed as Mean ± SD (n = 6). * *P* < 0.05 *vs*. Saline group; ^†^ *P* < 0.05 *vs*. UTMD alone group; ^#^ *P* < 0.05 *vs*. APS alone group.

The results of Masson staining analysis are shown in Figure 4, which demonstrated that neatly arranged myocardial cells, the nucleus and collagen fibers were shown to be dark and bright green, respectively. Only a small amount of distribution was observed in the myocardial interstitium and around the blood vessels of the healthy rats. In the DCM model group, the myocardial cells were hypertrophic, necrotic, and disorganized. Moreover, the bright green collagen fibers in the myocardial interstitium were significantly increased, and significant myocardial fibrosis were also observed. The disarrangement of myocardial cells in the combination treatment group was significantly improved, the bright green collagen fibers in myocardial interstitium were also significantly reduced as compared with saline-treated DCM ones, and the therapeutic effect was better than both two monotherapies.

### 3.5 Chronic treatment of combined APS microbubbles with UTMD improves inflammation and oxidative stress in DCM rats

The inflammation-related indicators, including IL-1β, TNF-α, and NF-κB, as well as oxidative stress-related factors, including SOD, MDA, and GSH-PX, in cardiac tissues were detected. As shown in Figure 4a-c, chronic treatment of combined APS microbubbles with UTMD significantly reduced the expression levels of all of the above indicators compared with the saline-treated DCM rats, indicating the current ameliorative inflammatory response in myocardium. In contrast, UTMD or APS alone treatment exhibited no significant change as compared with the model control ones. Moreover, as shown in Figure 4d-e, both the levels of SOD and GSH-PX in myocardial tissues from saline-treated DCM rats were both significantly decreased as compared to the normal rats, while the MDA level was significantly increased, suggesting the presence of oxidative stress. Chronic treatment of combined therapy reversed the decreased levels of MDA, SOD and GSH-PX and close to those of healthy rats, suggesting that the treatment of APS microbubbles combined with UTMD effectively improved the oxidative stress response accompanied by myocardial injury. The above results collectively revealed that the combined therapy could alleviate the inflammatory as well as oxidative stress response, thus protecting the damaged cardiac tissues of DCM rats.

### 3.6 Chronic treatment of combined APS microbubbles with UTMD activated the AMPK signaling pathway in DCM rats

**Figure 5.**
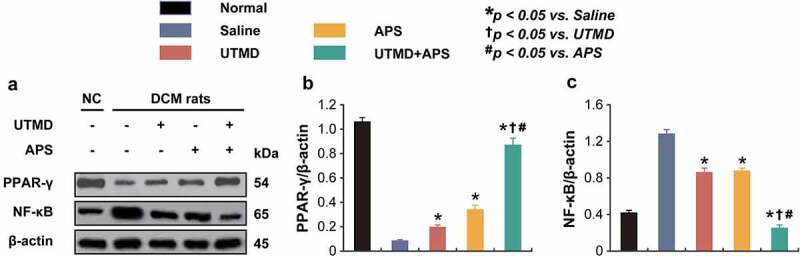
Effects of combined APS microbubbles with UTMD on the protein expression of PPAR-γ and NF-κB. The protein expression of (a) PPAR-γ and (b) NF-κB. All results showed as Mean ± SD (n = 3). * *P* < 0.05 *vs*. Saline group; ^†^ *P* < 0.05 *vs*. UTMD alone group; ^#^ *P* < 0.05 *vs*. APS alone group.

**Figure 6.**
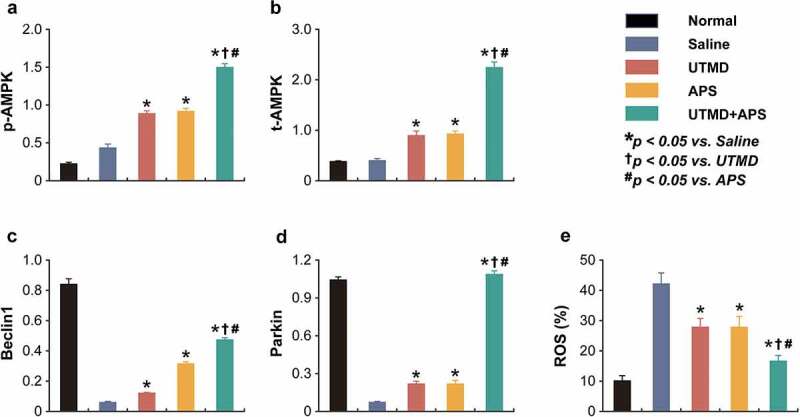
Chronic efficacies of combined APS microbubbles with UTMD on AMPK-dependent autophagic signaling in model rats. The protein relative expression of (a) p-AMPK, (b) t-AMPK, (c) Beclin1, (d) Parkin and (e) ROS content. All results showed as Mean ± SD (n = 3).* *P* < 0.05 *vs*. Saline group; ^†^ *P* < 0.05 *vs*. UTMD alone group; ^#^ *P* < 0.05 *vs*. APS alone group.

As shown in the Figures 5–6, the expression levels of p-AMPK and t-AMPK, as well as PPAR-γ and NF-κB in myocardial tissues of DCM or healthy rats were measured. Compared to DCM model rats, the expression levels of p-AMPK and t-AMPK in the group received combination therapy were significantly up-regulated (*P* < 0.05). Moreover, the protein expression level of PPAR-γ and NF-κB in myocardial tissues of DCM-received combination therapy were significantly increased and decreased, respectively, as compared with those of the saline-treated DCM ones, of which the enhanced improvement effects were also observed in both two monotherapy treated groups. In addition, combination therapy significantly increased the expression levels of Beclin1 and Parkin and reduced the ROS% level compared to those of saline-treated group and both two monotherapies. The above results collectively demonstrated that the combined APS microbubbles with UTMD could effectively reduce hyperglycemia-induced myocardial damages via by activating the up-regulation of AMPK and PPAR-γ signaling pathway, inhibiting the activity of NF-κB in myocardial tissues of DCM rats, thereby improving the damage.

## 4. Discussion

Previous studies have found that APS can significantly reduce blood glucose, free fatty acids and subsequently reduce the myocardial injury in diabetic rats [^18–21^]. In a rat model of type 2 diabetes constructed by diet combined with streptozotocin, the decrease in blood glucose was small after 4 weeks of APS intervention, but after 8 weeks of intervention, the effect of hypoglycemic effect was very significant, while lipid metabolism disorders that were significantly improved could be observed [22]. APS can prevent the development of lipotoxic cardiomyopathy by mainly relying on cardiac PPARα mechanisms [^23–26^]. Studies have shown that APS improves glucose and lipid metabolism and improves insulin resistance through the SIRT1-PGC-1α/PPARα-FGF21 signaling pathway [27]. Relevant studies have found that MMP-2 expression is reduced in cardiomyocytes of diabetic mice and promotes myocardial fibrosis [28]. It has also been shown that APS can reduce TGF-β1 and TNF-α expression, increase SOD activity, reduce MDA content, reduce myocardial oxidative stress and fibrosis, and reduce diabetes-induced myocardial injury in DCM rats [29].

Ultrasound targeted microbubble can selectively identify and accumulate in the target tissue, so as to achieve the purpose of targeted imaging and targeted therapy [13]. The technology has been rapidly developed in recent years [13]. At present, it is generally accepted that the acoustic pore effect is the main mechanism by which ultrasonic radiation destroys targeted microbubbles to achieve targeted drug release and gene transfection [30]. Acoustic pore effect refers to that the energy release generated when the microbubbles are cavitated can form shock waves, which act on the endothelial cell membrane to form shear stress so that temporary open holes appear on the membrane [27]. If this small hole belongs to a non-lethal acoustic hole, extracellular drugs and genes can be delivered into the cell at the moment of its opening to realize the transmission of targeted genes or drugs, and then achieve the purpose of targeted treatment of diseases [28]. Microbubble contrast agent, as a new type of transport carrier, can carry a variety of therapeutic active substances, can burst under a certain energy of ultrasound irradiation and accurately release active substances in the lesion, so as to achieve the purpose of targeted therapy [31,32].

It is worth mentioning that the rational construction of diabetic cardiomyopathy models is important to evaluate the therapeutic potential of candidate therapies. Adriamydine (ADR) is an anthraquinone anticancer antibiotic with a wide antitumor spectrum and strong anti-tumor activity, which is one of the most effective drugs for clinical treatment of solid tumors. Because the affinity of ADR to myocardial tissue is significantly higher than that of other tissues, it has severe cardiotoxicity. The acute toxic effects of ADR on the heart are mainly manifested as myocarditis and arrhythmia, while long-term use easily leads to dilated cardiomyopathy and congestive heart failure. Because the changes of myocardial structure and function induced by ADR in rats are most similar to the characteristics of human ADR cardiomyopathy, and the rat model is simple, economical and reproducible; therefore, the ADR-induced DCM rats were used in this study.

Combined with the results of many previous studies, we believe that APS holds the potential to be an option for the effective treatment of DCM. However, considering the conventional administration mode of APS makes it difficult to specifically enrich to heart. Therefore, we propose to combine ultrasound microbubble site-directed release technology to achieve effective targeted therapy, but it still needs to be systematically evaluated by rigorous animal *in vivo* experiments. In this study, we wished to investigate the potential preventive and therapeutic effects of APS combined with UTMD on diabetic cardiomyopathy in rats.

In this study, we prepared SonoVue microbubbles loaded with APS by non-covalent physical binding method and then used ultrasound energy to target and rupture the microbubbles in the damaged myocardial tissue of rats and release the therapeutic molecules to achieve the enrichment of APS in the myocardial tissue in a rat diabetic cardiomyopathy model, so as to investigate its improvement and therapeutic effect on DCM in an animal model. First, we observed and recorded the general diabetic parameters of DCM model rats during the experiment, including fasting blood glucose, glycosylated hemoglobin, and so on, and the above parameters of DCM model rats were significantly higher than those of healthy ones. After 6 weeks of continuous intervention with APS and APS combined with UTMD, these symptoms of DCM model rats were significantly improved as compared with the model control group, and there were no significant differences between the two APS treatment groups. After 6 weeks of continuous interventionr, the LVEF and LVFS indexes of rats in the APS and UTMD combination group were significantly higher than those in the DCM model group (all *P* < 0.05), while LVIDs and LVIDd were significantly smaller (*P* < 0.05). LVEF and LVFS were significantly higher in APS group than in UTMD group (all *P* < 0.05). Furthermore, the staining results of H&E and Masson staining suggested that the myocardial tissue injury was severe in the DCM model group 6 weeks later, and increased and disorganized myocardial interstitial collagen fibers and significantly enlarged cardiomyocytes with spaces could be observed, and vacuoles could be observed. Moreover, we measured the heart weight and the specific gravity of body weight in DCM model rats, and the above parameters of DCM model rats were significantly higher than those of healthy ones (*P* < 0.05), suggesting more severe myocardial hypertrophy. Combined with literature reports, long-term high glucose state can cause significant fibrosis in rat myocardium, which leads to more rigid ventricular wall and enlarged ventricular chamber, resulting in significantly worse cardiac function, including LVEF, LVFS, Sr, SRr values, in rats. In our study, we saw that the combination significantly improved the above symptoms and improved more significantly relative to APS alone.

As a vasodilator, CGRP can significantly relax the coronary vessels, and reduce blood viscosity and coronary vascular resistance, which can play a major role in regulating regional cardiac blood flow by increasing cardiac blood flow and oxygen delivery [33]. Endothelin, on the other hand, has a completely opposite regulatory effect on blood pressure and blood circulation than CGRP [33]. In the healthy state, the concentrations of CGRP and endothelin are in a balanced state, the secretion of the two substances is in a dynamic balance, the excessive release of endothelin will inhibit the secretion of calcitonin gene-related peptide, and the imbalance of its secretion will lead to the occurrence of diseases [33]. As the ‘gold standard’ for diagnosing myocardial injury, cTnI is highly specific [34]. Under long-term hyperglycemia, ET levels in myocardial tissue will continue to increase, while CGRP that antagonizes ET will be continuously consumed, which in turn causes cardiovascular contraction to decrease cardiac supply, further strengthens myocardial ischemia and hypoxia injury and in turn inhibits myocardial tissue to continue to secrete CGRP and enter irreversible myocardial damage [35].

It is very easy to understand that inflammatory factor release can promote the occurrence of inflammation and ultimately contributing to a continuous increase in ROS levels [22]. Previous reports have shown that NF-κB, as a key factor in regulating signaling pathway closely related to systemic inflammatory reactions inflammatory, participated in the transcription and modulation of many inflammatory mediators [36]. Reports have shown that the release of IL-1β and TNF-α, which could be promoted by activated NF-κB activation, stimulated the production of inflammatory factors then resulted in excessive hypertrophy of cardiomyocytes, and finally irreversible damage to myocardial tissue fibrosis [37]. In this study, we further found that long-term treatment of APS can significantly reduce the expression of all tested pro-inflammatory markers that lead to a notable reduction of inflammatory response in myocardial tissue, thereby significantly reducing the occurrence of inflammatory response, and has a significant advantage over APS alone.

SOD is an antioxidant metalloenzyme existing in organisms, which can catalyze the disproportionation of superoxide anion radicals to generate oxygen and hydrogen peroxide [38]; GSH-Px is an important peroxidolytic enzyme, which can protect the structure and function of cell membranes from peroxide interference and damage, and the balance of oxidation and anti-oxidation in vivo plays a crucial role [39]. MDA content is an important parameter reflecting the potential antioxidant capacity of the body, which can reflect the rate and intensity of peroxidation, and can also indirectly reflect the degree of tissue peroxidation damage [40]. The activities of SOD and GSH-PX in myocardial tissue of DCM model rats were significantly lower than those of healthy rats (*P* < 0.05), while the content of MDA was significantly higher (*P* < 0.05). After 6 weeks of continuous treatment intervention with APS combined with UTMD, the activities of SOD and GSH-PX in myocardial tissue increased, while the MDA content decreased significantly, with significant statistical differences relative to the model control group (all *P* < 0.05). In addition, the combination of APS and UTMD has a significant therapeutic advantage over APS alone, suggesting that the use of ultrasound energy loaded APS SonoVue microbubbles at the targeted rupture site of myocardial tissue can effectively enrich APS in myocardial tissue, thus playing a role in the treatment of myocardial tissue injury caused by high glucose.

As an energy sensor, AMPK plays a key role in the regulation of cellular energy balance, and is also gradually considered by the industry as a key modulator of cardiac energy metabolism [41]. Phosphorylated AMPK can participate in cardiac energy metabolism in vivo and can effectively reduce myocardial cell injury [41]. In the present study, the results of our *in vivo* experiments further demonstrated the significantly increased expression levels of the p-AMPK, Beclin1, and Parkin in the heart tissues of DCM model rats after 6 weeks of continuous intervention with APS combined with UTMD compared with saline-treated model control group, and the improvement was more significant relative to APS alone. The above data suggested that long-term administration of APS combined with UTMD can ultimately reduce cardiac injury in diabetic rats by significantly activating the AMPK signaling pathway, which in turn improves cardiomyocyte apoptosis.

## 5. Conclusion

This study demonstrated that the combination of ultrasound microbubbles loaded with APS and UTMD can effectively treat diabetic myocardial injury and protect the cardiac functions of DCM rats, and the mechanism may be related to the improvement of myocardial antioxidant capacity and slow the formation of myocardial fibrosis by the APS enriched in the heart. Current combination therapy is expected to be a new method for clinical treatment of DCM.

## Abbreviation

ultrasound-targeted microbubble destruction, UTMD; astragalus polysaccharide, APS; diabetic cardiomyopathy, DCM; Goto-Kakizaki, GK; left ventricular internal dimension, LVID; Left Ventricular Diastolic Dimension, LVIDD; left ventricular ejection fraction, LVEF; left ventricular fraction shortening, LVFS; glutathione peroxidase, GSH-Px; interleukin-1β, IL-1β; Adenosine monophosphate activated protein kinase, AMPK; tumor necrosis factor, TNF-α; Endothelin, ET; Calcitonin gene-related peptide, CGRP; Cardiac troponin I, CTnI; Creatine kinase, CK; lactate dehydrogenase, LDH; Superoxide dismutase, SOD; Malondialdehyde, MDA; Peroxisome proliferator-activated receptor, PPAR; Oral glucose tolerance test, OGTT; glycosylated hemoglobin, HbA1c;
